# The influence of self-concept on prosocial behavior in delinquent juveniles: a chain mediation analysis involving belief in a just world and self-control

**DOI:** 10.3389/fpsyg.2026.1775490

**Published:** 2026-04-29

**Authors:** Hongchang Teng, Jiaxin Guo, Hanwen Sheng, Yuehua Li

**Affiliations:** 1 College of Education, Ludong University, Yantai, China; 2 Specialized Education Research Center, Ludong University, Yantai, China; 3 School of Psychology, Northeast Normal University, Changchun, China; 4 Basic Teaching Department, Yantai Vocational College, Yantai, China

**Keywords:** belief in a just world, delinquent juveniles, prosocial behavior, self-concept, self-control

## Abstract

Prosocial behavior reflects social adaptation and rehabilitation in delinquent juveniles. However, the cognitive and regulatory mechanisms linking self-concept to prosocial behavior remain unclear. This study examined whether belief in a just world (BJW) and self-control mediate the association between self-concept and prosocial behavior. Mediation was tested both independently and sequentially. In the sequential mediation model, self-concept influenced BJW. BJW then influenced self-control. Self-control, in turn, influenced prosocial behavior. A sample of 387 delinquent juveniles from correctional centers in Shandong Province, China, completed the self-concept scale, the belief in a just world scale, the brief self-control scale, and the prosocial behavior scale. Pearson correlation and sequential mediation analyses (PROCESS Model 6) were conducted. Results indicated: (1) self-concept, BJW, self-control, and prosocial behavior correlated significantly and positively; (2) BJW and self-control each independently mediated the effect of self-concept on prosocial behavior; (3) the effect of self-concept on prosocial behavior was also sequentially mediated by BJW and then self-control. These findings clarify the cognitive-regulatory pathway from self-concept to prosocial behavior in delinquent juveniles and underscore the importance of these factors in rehabilitation.

## 1 Introduction

Prosocial behavior, defined as voluntary actions intended specifically to benefit others or society (Eisenberg et al., 2006a,b), is fundamental to adolescent socialization and development. Delinquent juveniles, a term encompassing youths who have engaged in illegal or antisocial behavior, often struggle to adapt following such conduct. For this population, promoting prosocial behavior—acts that reaffirm social norms and repair harm—is vital, as it facilitates relationship repair and social integration and reduces recidivism (Kong, 2017; Teng et al., 2018). Therefore, identifying determinants of prosocial behavior among delinquent juveniles is essential for effective rehabilitation and enhanced public safety.

Despite this, the majority of research on juvenile delinquency has prioritized negative traits, such as aggression and association with antisocial peers (Su and Yang, 2017). This focus frequently overlooks crucial positive psychological factors essential for rehabilitation (Kong, 2017). Recent scholarship advocates for greater attention to individual strengths. Prosocial behavior is increasingly recognized as a key positive outcome; it is associated with empathy (Li et al., 2025), positive interpersonal relationships (Nelson et al., 2016), and psychosocial adaptation (Wei et al., 2015). Moreover, prosocial behavior has been shown to reduce externalizing problems (Renouf et al., 2010) and support emotional development (Duan et al., 2022). Collectively, this evidence underscores the significance of prosocial development in interventions with delinquent juveniles.

Research on adolescent prosocial behavior predominantly addresses external contextual factors, such as parental practices (Hou et al., 2018) as well as internal individual factors, notably self-control (Gottfredson and Hirschi, 1990). However, the dynamic interactions between these factors and self-concept—particularly through specific worldviews and self-regulatory processes—are insufficiently investigated among delinquent juveniles. Clarifying these relationships is crucial for identifying cognitive-regulatory mechanisms applicable to intervention. Accordingly, the present study integrates cognitive and developmental frameworks and tests a chain mediation model of these pathways.

Adolescence is a critical stage in the development of self-concept. Self-concept is defined as an individual’s organized, cognitive evaluation of oneself (Hou, 1991). According to self-verification theory, individuals are motivated to align their behavior with their self-perceptions (Cauley and Tyler, 1989). For delinquent juveniles, this suggests that a positive self-concept can facilitate prosocial behavior. This association is also explained by Self-Determination Theory (Ryan and Deci, 2000), which suggests that a positive self-concept fulfills needs for autonomy and competence. These needs motivate valued behaviors. Erikson’s psychosocial theory further identifies identity versus role confusion as the central developmental task during adolescence (Erikson, 1968). Successful resolution promotes prosocial adjustment. Failure may result in maladaptive behavior. Thus, cultivating a positive self-concept is especially important for rehabilitation among delinquent juveniles.

Self-concept is linked to prosocial behavior through cognitive and self-regulatory mechanisms. Beck’s cognitive theory (1976) suggests that worldviews, such as the belief in a just world (BJW), function as cognitive mediators of behavioral outcomes. Self-regulation—the capacity to control impulses and actions—supports sustained prosocial behavior. The Cognitive-Affective Personality System (CAPS; Mischel and Shoda, 1995) integrates these concepts, positing that behavior results from interactions among self-schemas (self-concept), cognitive appraisals (notably BJW), and self-regulation. Within this framework, self-concept is proposed to influence prosocial behavior via its interconnection with BJW and self-regulation, linking cognitive structure to regulatory process.

Empirical research has linked self-concept to prosocial behavior across diverse populations. Cauley and Tyler (1989) demonstrated that children with a positive self-concept exhibited higher levels of cooperation. Studies by Hardy et al. (2014) and Hertz and Krettenauer (2016) found that self-concept predicts prosocial behavior in both adolescents and adults. Christner et al. (2022) observed that moral self-concept correlates with prosocial behavior, with emotion serving as a mediator of this association. In Chinese samples, research indicates that self-concept predicts prosocial behavior among college students (Zhen et al., 2018; Lei et al., 2015), and Li et al. (2025) found that self-concept in early adolescence predicts subsequent prosocial behavior, with gratitude as a mediator. Since college students and delinquent juveniles share an age range (11–25 years; Zhang, 2017), these findings may extend to the latter population.

Beck’s cognitive theory posits that positive self-concept shapes broader worldviews (Liu and Wang, 2017). Individuals with a negative self-concept may perceive the world as chaotic and unpredictable, which can undermine their BJW (Smith et al., 1996). Conversely, a positive self-concept is associated with stronger beliefs in a just world, as individuals project their perceived sense of order onto their environment (Dalbert, 1999). Empirical research indicates that self-concept clarity and core self-evaluation—traits related to positive self-concept—are significant predictors of BJW (Sun et al., 2024; Pan et al., 2025).

A strong BJW is posited to enhance self-control. Individuals with strong BJW see the world as orderly and believe effort is rewarded. This belief may reduce distress and conserve cognitive resources. BJW may also encourage prosocial behavior as an investment in maintaining a fair social order (Teng et al., 2018). According to the Conservation of Resources (COR) theory (Hobfoll, 1989), individuals seek to safeguard their resources. Reduced perceived threat of injustice supports self-control, which in turn promotes prosocial behavior. Empirical findings show that justice-related beliefs are linked to improved self-regulation and reduced impulsivity (Ke et al., 2025). Both personal and general BJW have been found to predict prosocial behavior through increased communal orientation (Guo et al., 2022). Social support is known to foster prosocial behavior by strengthening BJW (Wang et al., 2023). In juvenile correctional settings, research shows that educators’ BJW influences their attitudes and shapes inmates’ prosocial development (Levy and Reuven, 2018), underscoring BJW’s importance in these contexts.

Self-control, defined as the capacity to regulate impulses, emotions, and behaviors to achieve long-term goals or meet social expectations, is closely linked to prosocial behavior. Peterson’s (1982) internal control model suggests that prosocial actions require suppressing self-interest, which uses self-control resources. Liang Y. et al. (2019) and Liang Z. et al. (2019) found that self-control promotes prosocial behavior, especially when personal and collective interests clash. Many studies show self-control strongly predicts prosocial and cooperative behavior (Coenen et al., 2022; Erickson et al., 2024).

Drawing on CAPS, self-verification, Erikson’s, and COR theories, this study had three aims. First, we tested correlations between self-concept, BJW, self-control, and prosocial behavior among delinquent juveniles. Second, we examined BJW and self-control as independent mediators. Third, we tested whether self-concept first influences BJW, which then affects self-control, and finally leads to prosocial behavior, thus clarifying the sequential mediation pathway.

## 2 Method

### 2.1 Research hypothesis

This cross-sectional correlational study proposes a sequential mediation model: self-concept → BJW → self-control → prosocial behavior. We hypothesize that self-concept influences prosocial behavior both directly and indirectly. In the indirect path, self-concept influences BJW, BJW influences self-control, and self-control leads to prosocial behavior. We base this sequence on theories and findings suggesting that cognitive worldviews (BJW) shape regulatory capacities (self-control), which, in turn, guide prosocial actions.

First, the mediation by BJW and self-control is theoretically supported. According to moral identity theory, BJW strengthens moral identity, which increases motivation to adhere to moral standards. These standards require self-control for their enactment (Hardy et al., 2014). Thus, BJW fosters a belief in a fair world, leading individuals to value societal rules. This increased valuation of rules subsequently motivates self-control, which helps individuals respect norms and avoid disruptive actions (Bai et al., 2014; Sang et al., 2019). Empirical studies confirm this sequence, showing that BJW predicts self-control, which in turn inhibits deviant behavior (Sang et al., 2019). This evidence reinforces the proposed sequential mediation pathway from BJW to self-control to reduced deviant behavior.

Second, the sequence has a strong theoretical foundation. A positive self-concept acts as a stable self-schema, fostering BJW as a cognitive appraisal of the world. Viewing the world as fair and predictable reduces uncertainty and, according to COR theory (Hobfoll, 1989), conserves psychological resources. This resource conservation enables greater self-control, which in turn supports prosocial behavior that may require overriding self-interest. Thus, the cognitive and regulatory elements are closely linked in the proposed model.

Drawing on the integration of the CAPS theory, COR theory, Erikson’s Psychosocial Development Theory, and Bronfenbrenner’s Ecological Systems Theory, these theoretical frameworks collectively highlight the interactive effects between individual cognition and environmental adaptation. We argue that the sequential mediation pathway is not only a micro-level cognitive-regulatory process but also a reflection of delinquent juveniles’ adaptation to the social environment. Their positive self-concept (formed through resolving the identity crisis in adolescence) fosters a rational BJW (a cognitive adaptation to the social system), which, in turn, conserves psychological resources for self-control and emotional regulation—core capacities for coping with social risks and for enacting prosocial behavior. We thus hypothesize a chain of mediation in which self-concept promotes a stronger BJW, which, in turn, enhances self-control, ultimately leading to more frequent prosocial behavior.

*H1*: Self-concept significantly and positively predicts prosocial behavior among delinquent juveniles.

*H2*: BJW mediates the relationship between self-concept and prosocial behavior.

*H3*: Self-control mediates the relationship between self-concept and prosocial behavior.

*H4*: BJW and self-control sequentially mediate the relationship between self-concept and prosocial behavior (self-concept → BJW → self-control → prosocial behavior).

### 2.2 Participants

We used a cross-sectional design with convenience sampling. Data were collected between September and November 2022 from juvenile correctional centers in Shandong Province, China. A total of 448 individuals were invited to participate, and 387 valid questionnaires were retained (effective response rate = 86.38%).

#### 2.2.1 Inclusion criteria

(1) aged 14–24 years; (2) formally detained or imprisoned due to criminal offenses; (3) no history of severe mental illness or cognitive impairment (assessed via self-report and facility records); (4) voluntary participation with informed consent.

#### 2.2.2 Sample characteristics

Mage = 17.74 years (SD = 2.11, range = 14–24); 297 males (76.7%) and 90 females (23.3%); predominantly Han Chinese (98.2%); 72.1% from biparental families, 27.9% from single-parent or reconstituted families; offenses included theft (41.3%), assault (28.7%), robbery (15.2%), and other non-violent offenses (14.8%). No significant differences in age, gender, offense type, and family structure were observed between valid and excluded participants (*p* > 0.05).

### 2.3 Instruments

The scales adopted in this study were developed by the National Collaborative Group for the Survey on the Psychological Developmental Characteristics of Chinese Children and Adolescents (Dong and Lin, 2011). Led by Professors Qi Dong and Chongde Lin from the State Key Laboratory of Cognitive Neuroscience and Learning at Beijing Normal University, this survey represents China’s first large-scale research project on the psychological developmental characteristics of Chinese children and adolescents. The research team developed a set of psychological development assessment tools suitable for Chinese adolescents, which cover four domains: cognitive ability, academic achievement, social adaptation, and developmental environment. All four scales used in the present study fall within the domain of social adaptation; they are self-report measures and have been validated with good psychometric properties in Chinese samples. Although the four scales use a 4-point response format rather than the 6-point or 7-point scales commonly adopted in international research, they assess the same core constructs and are conceptually comparable with similar scales used worldwide.

#### 2.3.1 Self-concept scale

The Scale includes 18 items across four dimensions: academic self, physical self, interpersonal self, and sports self. Responses were rated on a 4-point Likert scale (1 = completely inconsistent, 4 = completely consistent), with higher scores indicating a more positive self-concept. Items 1, 4, 5, 6, 11, and 15 are reverse-scored. Statistical analysis on a valid sample of 22,797 participants revealed that the overall Cronbach’s Alpha coefficient of the scale was 0.84, with the Cronbach’s Alpha coefficients for each dimension ranging from 0.74 to 0.84. Confirmatory factor analysis (CFA) conducted on a valid sample of 24,013 participants showed that the model fit indices were satisfactory, with all item factor loadings ranging from 0.42 to 0.85. The scale thus meets psychometric criteria and demonstrates good construct validity. The overall Cronbach’s Alpha was 0.81 in the current study.

#### 2.3.2 Belief in a just world scale

The Scale includes 13 items across two dimensions: general BJW and personal BJW. Responses were rated on a 4-point Likert scale (1 = Strongly Disagree, 4 = Strongly Agree), with higher scores indicating stronger BJW. Statistical analyses of the valid data from 23,377 participants indicated that the overall Cronbach’s alpha coefficients for the scale and its subdimensions ranged from 0.65 to 0.82. Confirmatory factor analysis (CFA) performed on an independent, valid sample of 24,013 participants indicated that the model fit indices were satisfactory. All item factor loadings ranged from 0.37 to 0.67, meeting psychometric standards and demonstrating good construct validity for the scale. The overall Cronbach’s Alpha was 0.82 in the current study.

#### 2.3.3 Brief self-control scale

The Scale comprises 5 items across a single dimension. Responses were rated on a 4-point Likert scale (1 = completely mismatch, 4 = completely match), with higher scores indicating better self-control. Statistical analyses of the valid data from 23,885 participants indicated that the scale’s overall Cronbach’s alpha was 0.58. Confirmatory factor analysis (CFA) performed on an independent, valid sample of 24,013 participants indicated that the model fit indices were satisfactory. All item factor loadings ranged from 0.34 to 0.58, meeting psychometric standards and demonstrating good construct validity for the scale. The overall Cronbach’s Alpha was 0.57 in the current study.

#### 2.3.4 Prosocial behavior scale

The Scale comprises 12 items across a single dimension. Responses were rated on a 4-point Likert scale (1 = never, 4 = always), with higher scores indicating more frequent prosocial behavior. Statistical analyses of the valid data from 23,963 participants indicated that the scale’s overall Cronbach’s alpha was 0.85. Confirmatory factor analysis (CFA) performed on an independent, valid sample of 24,013 participants indicated that the model fit indices were satisfactory. All item factor loadings ranged from 0.48 to 0.66, meeting psychometric standards and demonstrating good construct validity for the scale. The overall Cronbach’s Alpha was 0.78 in the current study.

### 2.4 Procedure

This study was conducted in accordance with the Declaration of Helsinki (World Medical Association, 2013) and approved by the Institutional Review Board of Ludong University. Prior to data collection, permission was obtained from correctional authorities.

Participants aged under 18 years provided written informed consent with their legal guardians, and those aged 18 years and above provided independent written informed consent. All participants were informed of the voluntary, anonymous nature of the study and their right to withdraw at any time.

Trained research assistants administered questionnaires in group settings within correctional facilities. Participants completed questionnaires independently, with neutral guidance provided for participants with reading difficulties. To minimize social desirability bias, participants were assured that responses would not affect their institutional status. Two correctional officers were on-site to maintain order during the questionnaire completion process.

Questionnaires were excluded based on the following criteria: (1) the missing data rate of core variables exceeded 10%; (2) responses showed obvious patterning or contradictions; (3) the completion time was less than 15 min (Before formal data collection, a pilot test was conducted with N = 36 participants from the same population. The lower 5% cutoff was approximately 15 min).

### 2.5 Data analysis

SPSS 21.0 was used for descriptive statistics and Pearson’s correlation analysis. The sequential mediation model was tested using Model 6 of the PROCESS macro for SPSS (Hayes, 2013), using the Bootstrap method (5,000 resamples, 95% confidence interval (95% CI)] to test the significance of mediation effects [a mediation effect was considered significant if the 95% CI did not include zero). Given the limitations of the available data, gender and age were included as control variables in the model to control for potential confounding effects of basic demographic characteristics.

## 3 Results

### 3.1 Common method bias test

Given that all data in this study were collected via self-reported questionnaires, common method bias (CMB) may be present. To address this issue, Harman’s single-factor test was conducted to examine potential CMB. In Harman’s single-factor test, the first extracted factor accounted for 14.19% of the total variance, which was well below the widely accepted critical threshold of 40%, and the total explained variance reached 72.4%, indicating no severe common method bias in this study.

The Cronbach’s Alpha coefficient for the Brief Self-Control Scale in this study was 0.57, which is below the conventional acceptable level of > 0.70. Low reliability may lead to attenuation bias and underestimate the true associations between variables. All results are interpreted with this limitation in mind.

### 3.2 Correlation analysis

Pearson correlation analysis was performed on the mean scores of self-concept, BJW, self-control, and prosocial behavior. As shown in Table 1, the results revealed significant positive correlations among all four variables (all *p* < 0.01). Self-concept was positively correlated with BJW (*r* = 0.18, *p* < 0.01), self-control (*r* = 0.25, *p* < 0.01), and prosocial behavior (*r* = 0.38, *p* < 0.01); BJW was positively correlated with self-control (*r* = 0.42, *p* < 0.01) and prosocial behavior (*r* = 0.27, *p* < 0.01); Self-control was positively correlated with prosocial behavior (*r* = 0.32, *p* < 0.01). These correlation results provided preliminary empirical support for the subsequent mediation effect analysis.

**Table 1:** Descriptive statistical results and correlation analysis between variables (*n* = 387).

	*M*	SD	Variable 1	Variable 2	Variable 3	Variable 4
1 Self-concept	2.66	0.40	—			
2 Belief in a just world	2.76	0.48	0.18^***^	—		
3 Self-control	2.65	0.52	0.25^***^	0.42^***^	—	
4 Prosocial behavior	2.51	0.46	0.38^***^	0.27^***^	0.32^***^	—

***p < 0.001 (two-tailed); M, mean; SD, standard deviation.

### 3.3 Sequential mediation model verification

The distribution of the key variables (self-concept, BJW, self-control, and prosocial behavior) was assessed using Skewness, kurtosis, and the Shapiro–Wilk test. The results showed that all variables were normally distributed (*p* > 0.05).

To test the proposed sequential mediation model, we conducted a mediation analysis using Model 6 of the PROCESS macro for SPSS, with gender and age included as control variables. Self-concept served as the predictor variable, prosocial behavior as the outcome variable, and BJW and self-control as the mediating variables. The Bootstrap method was employed to estimate and test the significance of mediation effects, with 5,000 resamples and a 95% confidence interval (CI); a non-zero 95% CI indicated a significant mediation effect.

#### 3.3.1 Regression analysis results

As presented in Table 2, the regression analysis results (After including age, gender, offense type and family structure, both the AIC and BIC values of the model decreased, indicating an improvement in model fit and stability.) showed that: (1) self-concept positively predicted BJW (*β* = 0.18, *p* < 0.001) and self-control (*β* = 0.17, *p* < 0.001) directly; (2) BJW exerted a direct positive predictive effect on self-control (*β* = 0.40, *p* < 0.001); (3) when BJW and self-control were included in the model, self-concept (*β* = 0.31, *p* < 0.001), BJW (*β* = 0.12, *p* < 0.05), and self-control (*β* = 0.21, *p* < 0.001) all significantly and positively predicted prosocial behavior. These results confirmed the preliminary conditions for the existence of mediation effects.

**Table 2:** Regression analysis of variable relationships in the chain mediation model.

Dependent variable	Predictors	*R*	*R^2^*	*F*	*β*	*t*
Belief in a just world		0.18	0.03	4.50**		
	Age				0.03	0.45
	Gender				0.04	0.60
	Offense type				−0.04	−0.60
	Family structure				0.06	0.82
	Self-concept				0.18	3.55***
Self-control		0.49	0.24	29.73***		
	Age				−0.11	−1.92
	Gender				−0.10	−1.90
	Offense type				0.07	0.95
	Family structure				0.05	0.76
	Self-concept				0.17	3.63***
	Belief in a just world				0.40	8.69***
Prosocial behavior		0.48	0.23	23.10***		
	Age				−0.02	−0.36
	Gender				0.16	2.89**
	Offense type				0.06	0.83
	Family structure				0.10	1.90
	Self-concept				0.31	6.63***
	Belief in a just world				0.12	2.49*
	Self-control				0.21	4.08***

All variables were standardized; *p < 0.05, **p < 0.01, ***p < 0.001 (two-tailed).

#### 3.3.2 Mediation effect analysis

**Figure 1:**
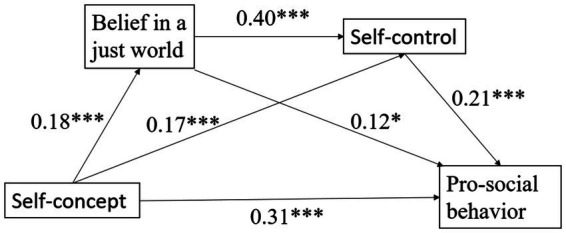
A chain mediation model. Gender and age were included as control variables (coefficients not shown); *p < 0.05, ***p < 0.001; solid lines indicate significant paths.

The mediation effect analysis results (see Table 3 and Figure 1) indicated that the total mediation effect of BJW and self-control was significant, with a value of 0.07. Specifically, the total mediation effect comprised three distinct indirect paths, all of which were significant (95% CI did not include zero).

**Table 3:** Bootstrap 95% confidence interval for the mediating effect path.

Mediation paths	Effect size	95% CI lower	95% CI upper	Relative mediation effect (%)
Total indirect effects	0.07	0.034	0.115	18.85%
Indirect path 1 (self-concept → BJW → prosocial behavior)	0.02	0.002	0.047	5.84%
Indirect path 2 (self-concept → self-control → prosocial behavior)	0.03	0.011	0.066	9.13%
Indirect path 3 (Self-concept → BJW → self-control → prosocial behavior)	0.01	0.004	0.029	3.88%

CI, confidence interval; relative mediation effect = (indirect effect/total effect) × 100%; relative mediating effect percentages were calculated using standardized coefficients.

*Indirect Path 1*: Self-concept → BJW → Prosocial behavior. The indirect effect was 0.02 [95% CI (0.004, 0.036)], confirming the significant independent mediating role of BJW, supporting H2.

*Indirect Path 2*: Self-concept → Self-control → Prosocial behavior. The indirect effect was 0.03 [95% CI (0.008, 0.052)], indicating a significant independent mediating role for self-control, supporting H3.

*Indirect Path 3 (Sequential Mediation Path)*: Self-concept → BJW → Self-control → Prosocial behavior. The indirect effect was 0.01 [95% CI (0.002, 0.020)], indicating that BJW and self-control exerted a significant sequential mediating effect on the relationship between self-concept and prosocial behavior, supporting H4.

Crucially, even after accounting for these indirect pathways, self-concept retained a significant direct effect on prosocial behavior (*β* = 0.31, *p* < 0.001), supporting H1. This suggests that while BJW and self-control are key mechanisms, other factors also contribute to translating self-concept into action. Collectively, these results support the proposed sequential mediation model.

## 4 Discussion

This study tested a sequential mediation model to explore the cognitive and regulatory mechanisms linking self-concept, confirming that self-concept exerted both direct positive effects on prosocial behavior and significant indirect effects via the independent and sequential mediating roles of BJW and self-control. These findings not only enrich the empirical evidence on positive psychological factors underlying prosociality in correctional populations but also construct a clear cognitive-regulatory pathway grounded in integrated theoretical frameworks, providing targeted theoretical support for juvenile rehabilitation practices.

### 4.1 Direct effect of self-concept on prosocial behavior

Consistent with Hypothesis 1 and self-verification theory, self-concept emerged as a significant positive predictor of prosocial behavior, which aligns with mainstream empirical findings in adolescent developmental research (Cauley and Tyler, 1989; Hardy et al., 2014). Unlike the general adolescent population, delinquent juveniles often carry persistent negative self-perceptions due to past deviant behaviors and correctional experiences, which easily lead to self-denial and social withdrawal. According to self-verification theory, individuals tend to exhibit behaviors consistent with their core self-views to maintain identity coherence; for delinquent juveniles, a positive self-concept can break the cycle of negative self-cognition linked to delinquency, rebuild their sense of self-worth and social belonging, and further drive voluntary prosocial acts that conform to social norms.

In addition, this study found significant gender differences in the prosocial behavior of delinquent juveniles, a result that is consistent with mainstream research findings in the field of adolescent prosocial behavior and aligns with the developmental and correctional characteristics of this special adolescent population. From the perspective of gender role socialization, traditional gender role norms hold softer, empathetic, altruistic, and cooperative expectations for females. During adolescence, females tend to receive earlier guidance and reinforcement for prosocial behaviors and are more inclined to prioritize others’ needs and suppress self-interested impulses, thereby exhibiting higher levels of prosocial behavior.

### 4.2 Independent mediating roles of BJW and self-control

The significant independent mediating effect of BJW verified Hypothesis 2, which is fully consistent with Beck’s cognitive theory (Beck, 1976; Liu and Wang, 2017) and the COR theory (Hobfoll, 1989). Beck’s cognitive theory holds that individual self-cognition shapes external-world cognition, and a positive self-concept helps delinquent juveniles eliminate biased perceptions of the external environment as unfair and chaotic, and form a rational BJW. From the perspective of COR theory, a high level of BJW reduces the psychological pressure and cognitive resource loss associated with perceived injustice and resentment, providing a stable cognitive basis for positive behavioral choices. For delinquent juveniles, BJW makes them believe that their prosocial efforts will be fairly perceived and rewarded, thereby strengthening their willingness to engage in prosocial behaviors and bridging the gap between positive self-cognition and actual prosocial acts (Teng et al., 2018).

The independent mediating effect of self-control supported Hypothesis 3, which echoes classic theoretical viewpoints such as the altruism internal control model (Peterson, 1982) and COR theory (Hobfoll, 1989). Prosocial behavior often requires individuals to suppress immediate self-interested impulses and to consider collective or others’ interests, which relies heavily on sufficient self-control resources (Liang Y. et al., 2019; Liang Z. et al., 2019). Positive self-concept reduces internal psychological conflicts and anxiety in delinquent juveniles, effectively conserving their psychological and regulatory resources, thereby enhancing self-control ability. The strengthened self-control further helps them resist impulsive and antisocial tendencies and stably implement prosocial behaviors. This finding reveals the important regulatory role of self-control in the behavioral transformation of delinquent juveniles, and confirms that self-control is a key internal factor connecting positive cognition and adaptive behavior.

### 4.3 Sequential mediating role of BJW and self-control

The present study provides preliminary support for Hypothesis 4, suggesting that BJW and self-control may function as a sequential mediating chain in the relation between self-concept and prosocial behavior. This pathway is highly consistent with the CAPS theory (Mischel and Shoda, 1995), which emphasizes that individual behavior is the dynamic interaction outcome of self-schemas, cognitive appraisals, and regulatory competencies. Specifically, positive self-concept (self-schema) first promotes the formation of BJW (positive cognitive appraisal of the world), and BJW, as a vital cognitive resource, further reduces environmental uncertainty and negative emotional arousal, conserving regulatory resources for self-control (Bai et al., 2014; Sang et al., 2019); finally, enhanced self-control transforms internal positive beliefs and motivations into concrete prosocial behaviors.

This sequential mediation model addresses the limitations of single-factor or parallel mediation models in previous research, revealing a progressive, interactive cognitive-regulatory mechanism. Different from simply examining the independent effects of single variables, this study clarifies the logical sequence of “positive self-cognition → rational world belief → behavioral regulation ability → prosocial behavior”, which deepens the understanding of the internal formation mechanism of prosocial behavior in delinquent juveniles. Meanwhile, this chain pathway also reflects the systemic nature of individual psychological and behavioral development, indicating that the cultivation of prosocial behavior in correctional practice cannot rely on a single intervention, but requires systematic guidance across the entire cognitive-regulatory process.

### 4.4 Practical implications

The practical implications of this study are directly informed by empirical findings and tailored to juvenile correctional settings. Recommendations focus on targeted, actionable strategies addressing self-concept, BJW, and self-control, as identified in the sequential mediation model.

#### 4.4.1 Self-concept positivity enhancement: targeted positive cognitive counseling

In view of the prominent negative self-perception of delinquent juveniles, correctional institutions should carry out short-term, targeted positive cognitive group counseling, focusing on guiding them to re-examine themselves, discover personal advantages and positive qualities, and get rid of negative labels caused by deviant behaviors. Combined with the direct effect of self-concept on prosocial behavior found in this study, counseling can adopt self-affirmation training and positive experience sharing, helping them gradually establish a positive, stable self-concept and lay a cognitive foundation for the generation of prosocial motivation.

#### 4.4.2 BJW cultivation: rational attribution and fair perception training

Based on the mediating role of BJW, as verified in this study, targeted attribution training should be provided to delinquent juveniles to correct their negative attribution bias that “all misfortunes are caused by an unfair world.” Psychological counselors can use the daily rules and fair management practices of correctional institutions to help them correctly understand the relationship between personal effort and behavioral outcomes, and gradually establish a rational and positive BJW. This can enhance their recognition of social norms and fairness, and further stimulate their willingness to practice prosocial behavior.

#### 4.4.3 Self-control improvement: behavioral regulation training integrated with cognitive guidance

Combined with the final mediating role of self-control in the chain pathway, correctional institutions can implement simple, feasible self-control training, such as impulse-control exercises and delayed-gratification activities, targeting the characteristic that delinquent juveniles are prone to impulsive behavior. Notably, recent research on Chinese male delinquent juveniles shows that social support can enhance self-control and reduce aggression through trait mindfulness (Ke et al., 2025), which supports the effectiveness of integrating regulatory training with cognitive-behavioral interventions in this study. Relying on the conclusion that BJW can promote self-control, training should be linked with the cultivation of fair belief, so that self-control training is not an isolated behavioral exercise, but a regulatory ability improvement based on positive cognitive change, so as to ensure the sustainability of intervention effects and effectively promote the transformation of prosocial motivation into actual behavior. In addition, relevant research has verified that targeted self-control intervention programs are feasible and effective in juvenile correctional settings (Tcherni-Buzzeo, 2023; Erickson et al., 2024), providing further empirical support for the practical implications proposed in this study.

### 4.5 Limitations and future directions

This study has several limitations that need to be addressed in future research. First, the cross-sectional design precludes definitive causal conclusions; longitudinal or experimental designs are needed to verify the temporal order of variables. Second, exclusive reliance on self-report measures may introduce response bias; future research should incorporate multi-method assessments (e.g., behavioral tasks, staff ratings). Third, the sample was drawn exclusively from Shandong Province, limiting generalizability to other cultural or correctional contexts. Fourth, the Brief Self-Control Scale demonstrated low internal consistency in our sample, which may have attenuated the observed correlations; future studies should employ more robust measures of self-control with this population.

Future research should address these limitations through longitudinal designs, multi-method assessments, and more diverse samples. Additionally, exploring moderating variables (e.g., moral identity, social support) and developing targeted interventions based on the sequential pathway would further advance this field. From the perspective of Bronfenbrenner’s Ecological Systems Theory, future research can also examine cross-level interactions between individual cognitive factors and environmental systems on prosocial behavior. Relevant recent studies have provided differentiated directions for subsequent targeted interventions and in-depth exploration (Vlckova and Burianek, 2025; Boonmann et al., 2023).

Of particular note, the Cronbach’s Alpha coefficient of the Brief Self-Control Scale in the present study was 0.57, which falls considerably below the commonly accepted threshold of 0.70. Inadequate reliability of the scale may introduce measurement error and attenuation bias, potentially leading to an underestimation of true associations among variables and thereby affecting the precision and robustness of the present findings. To enhance measurement accuracy and reliability, future research may employ larger, more homogeneous samples that better align with the scale’s normative population or adopt alternative instruments for assessing self-control with superior psychometric properties. The small effect sizes observed in this study suggest weak practical significance. Conclusions should be interpreted with caution. Future studies with improved designs, larger samples, and better measurement methods may help verify the stability and magnitude of these effects.

## 5 Conclusion

This study preliminarily supports the sequential mediation model, indicating that positive self-concept may predict BJW, which, in turn, may contribute to self-control capacity and ultimately promote prosocial behavior. In other words, BJW and self-control may sequentially mediate the relationship between positive self-concept and prosocial behavior. The indirect effects identified in this study are relatively weak, and the relative contributions of each path are modest.

Based on classical theories, including the Cognitive-Affective Personality System Theory, Conservation of Resources Theory, and Self-Verification Theory, the present findings provide preliminary empirical clues and theoretical implications for understanding the mechanisms underlying prosocial behavior among delinquent juveniles. These results may enrich relevant research on positive psychology and behavioral development in this population. Further replication across diverse samples and groups is needed to confirm the generalizability and robustness of the present findings.

## Data Availability

The raw data supporting the conclusions of this article will be made available by the authors, without undue reservation.
