# Supplementary material for: The influence of self-concept on prosocial behavior in delinquent juveniles: a chain mediation analysis involving belief in a just world and self-control
Source: Front Psychol. 2026 Apr 29;17:1775490. doi: 10.3389/fpsyg.2026.1775490 (PMC13168102; doi:10.3389/fpsyg.2026.1775490)
Supplement: Supplementary file 1 [file Supplementary_file_1.doc]

**Appendix: Detailed Items of Instruments**

This appendix provides the full item lists, response formats, and scoring rules for all scales used in the study, for readers to access and verify. All scales are translated from the original Chinese version, ensuring consistency with the original meaning and adaptability to academic expression.

**1. Self-Concept Scale**

Response Format: 4-point Likert scale (1 = Completely Inconsistent, 2 = Inconsistently, 3 = Basically Consistent, 4 = Completely Consistent). Higher scores indicate a higher degree of consistency between the item description and the respondent’s actual situation. There is no right or wrong answer; please select only one option per item.

Full Items:

1. It is difficult for me to make friends with others.

2. I like activities such as sports, gymnastics and dance.

3. My face looks good.

4. I get poor grades in most courses.

5. I have few friends.

6. Not many classmates like me.

7. Most courses are too difficult for me.

8. My figure looks good.

9. I am better than most of my friends in activities such as sports, gymnastics and dance.

10. I can easily make friends with others.

11. I hate activities such as sports, gymnastics and dance.

12. Others think I look good.

13. I do well in most courses.

14. All classmates like me.

15. I seem stupid in the study of most courses.

16. I get good grades in most courses.

17. I look good.

18. I perform better than other classmates in activities such as sports, gymnastics and dance.

**2. Belief in a Just World Scale (BJW)**

Response Format: 4-point Likert scale (1 = Strongly Disagree, 2 = Slightly Disagree, 3 = Basically Agree, 4 = Strongly Agree). Higher scores indicate a stronger agreement with the statement. There is no right or wrong answer; please select only one option per item.

Full Items:

1. I believe that justice will eventually prevail over injustice.

2. There will always be injustice in life, but I firmly believe this is only an exception, not the norm.

3. I think people will try their best to make important decisions fairly.

4. I believe that in this society, good people will be rewarded and bad people will be punished.

5. I am convinced that people who suffer injustice will eventually be compensated.

6. I think the world is a fair place.

7. Overall, the things that happen to me in life are fair.

8. I am usually treated fairly.

9. I believe I get what I deserve.

10. I believe that if I do good things, I will get good rewards; if I do bad things, I will get bad punishments.

11. The unjust things that happen to me in life are only exceptions, not the norm.

12. I believe most of the things that happen to me in life are fair.

13. I think the important decisions about me are usually fair.

**3. Self-Control Scale**

Response Format: 4-point Likert scale (1 = Completely Inconsistent, 2 = Inconsistently, 3 = Basically Consistent, 4 = Completely Consistent). Higher scores indicate a higher degree of consistency between the item description and the respondent’s actual daily situation. There is no right or wrong answer; please select only one option per item.

Full Items:

1. I can control my feelings even when dealing with things I hate or dislike.

2. I will eat food I don’t like for the sake of health.

3. I won’t show my anger on my face even when I am angry.

4. I won’t overeat food I like if it is bad for my health.

5. When I know something is bad, I will restrain myself from doing it.

**4. Prosocial Behavior Scale**

Response Format: 4-point Likert scale (1 = Never, 2 = Sometimes, 3 = Often, 4 = Always). Higher scores indicate a higher frequency of the described behavior. There is no right or wrong answer; please select only one option per item.

Full Items:

1. I will abide by various school rules and regulations.

2. I like to participate in social welfare activities organized inside and outside the school.

3. When others cannot do something well, I will encourage them.

4. When I see others in trouble, I will take the initiative to help them.

5. I am happy to donate money and materials to disaster areas.

6. When participating in school activities (such as playing ball games), I cooperate with my teammates tacitly.

7. I will take the initiative to invite onlookers to join our games.

8. I will help classmates make up lessons or teach them to play ball games.

9. When classmates need it, I am willing to let them use my things.

10. I am willing to do things for the class collective.

11. I will tolerate classmates who make mistakes.

12. I will keep my promises.
